# Intermediate PSA half-life after neoadjuvant hormone therapy predicts reduced risk of castration-resistant prostate cancer development after radical prostatectomy

**DOI:** 10.1186/s12885-017-3775-6

**Published:** 2017-11-23

**Authors:** Yong Jin Kang, Won Sik Jang, Jong Kyou Kwon, Cheol Yong Yoon, Joo Yong Lee, Won Sik Ham, Young Deuk Choi

**Affiliations:** Department of Urology, Urological Science Institute, Yonsei University College of Medicine, 50-1 Yonsei-ro, Seodaemun-gu, Seoul, Republic of Korea

**Keywords:** Prostate, Neoadjuvant androgen deprivation therapy, Prostate-specific antigen

## Abstract

**Background:**

The magnitude and rapidity of the tumor response to androgen deprivation is known to predict the durability of the therapy. We have investigated the predictive value of categorizing patients by the half-life of PSA under neoadjuvant androgen deprivation therapy in patients with biochemical recurrence after radical prostatectomy.

**Methods:**

Medical records of 317 patients who received neoadjuvant androgen deprivation therapy before radical prostatectomy and developed biochemical recurrence were analyzed. The patients were categorized into five groups according to PSA half-life. Risk of developing castration resistance was evaluated by Kaplan-Meier analysis and by Cox proportional risk regression analysis.

**Results:**

The median follow-up duration was 50.1 months (IQR 31.8–68.7) and median PSA half-life was 22.1 days (IQR 12.7–38.4). Comparison of survival curves revealed that patients in the intermediate response group showed significantly lower 5-year castration-resistant prostate cancer rate (37.5%) compared to non-response and ultra-rapid response groups (63.6%, *p* = 0.007; 56.1%, *p* = 0.031; respectively). In the multivariate regression model, intermediate response compared to non-response was associated with significantly reduced risk of castration resistance development (hazard ratio 0.397, 95% confidence interval 0.191–0.823, *p* = 0.013) and overall mortality (hazard ratio 0.138, 95% confidence interval 0.033–0.584, *p* = 0.007). When subcategorized by Gleason score, Kaplan-Meier curve revealed that, in the high Gleason score stratum, 5-year castration-resistant prostate cancer rate for intermediate response group (44.0%) was exceptionally lower than that in non-response group (66.7%, *p* = 0.047), while castration resistance increased in other groups.

**Conclusion:**

Short PSA half-life as well as no response after androgen deprivation is associated with increased risk of treatment failure compared to intermediate PSA half-life.

**Electronic supplementary material:**

The online version of this article (10.1186/s12885-017-3775-6) contains supplementary material, which is available to authorized users.

## Background

Androgen deprivation therapy (ADT) has been the preferred therapy for prostate cancer patients with biochemical recurrence (BCR) after definitive local therapy. Despite the initial tumor suppressing effect, the majority of patients develop resistance to medical castration within 2–3 years, rendering the ADT ineffective [1–3]. Modification of the androgen receptor is believed to be the most important cause underlying castration-resistant prostate cancer (CRPC) development, which enables androgen-independent activation of the androgen receptor or alters its sensitivity [1]. Nevertheless, it is known that the androgen axis continues to play an important role in the function and growth of CRPC [1, 4].

Regarding the risk of developing CRPC, there has been a notion that the magnitude [5] and rapidity [6] of the tumor response to ADT can reliably predict the durability of the therapy. Some prior studies suggest that a shorter time to nadir or prostate-specific antigen half-life (PSAT_½_) during ADT is associated with longer CRPC-free survival and cancer-specific survival [7, 8]. However, there have been some recent studies with contradictory results, reporting shorter remission period and poorer survival in rapid response groups undergone ADT [9, 10]. Because tumor response to ADT may vary [11] and some prostate cancer cells are known to produce little, if any, prostate-specific antigen (PSA) [12], the resultant PSA kinetics can be heterogeneous in nature [3].

Under the assumptions that the risk of developing CRPC can be measured by analyzing the PSA response to hormone therapy and that castration resistance is heterogeneous among the individual tumor cells within a patient, we investigated the value of the PSAT_½_ during neoadjuvant ADT as a predictive factor for CRPC in radical prostatectomy patients who developed BCR.

## Methods

### Patient population

With approval from the Severance Hospital Institutional Review Board (protocol number 4–2016-0506), the clinical information and follow-up data of patients who underwent bilateral nerve-sparing radical prostatectomy in a single center by a single operator (Y.D.C.) between 2002 and 2014 were collected. Informed consent from the participants was waived by the institutional review board. Patients who received neoadjuvant ADT and developed BCR (*n* = 348) were selected. Patients with missing data (*n* = 18) or those who received adjuvant radiotherapy prior to or on the day of BCR diagnosis (*n* = 13) were excluded from the cohort, leaving 317 patients for analysis. No patients were given cytotoxic chemotherapy before the confirmation of CRPC.

### Neoadjuvant therapy and preoperative parameters

Neoadjuvant hormone therapy in the study cohort consisted of bicalutamide monotherapy (50 mg bid), administered based on treating physician’s decision. Regression due to neoadjuvant therapy was noted on most of the specimens, and in 7 (2%) patients the regression was so extensive that there were no tumor cells detectable despite the positive results on preoperative biopsy. Median duration of neoadjuvant ADT was 53 (interquartile range [IQR] 35–106) days. Initial PSA was measured at the time of screening immediately before the transrectal ultrasound-guided prostate biopsy. Preoperative serum PSA levels were sampled the day before the operation.

PSAT_½_ was calculated by the following formula [13]:

$$ \mathrm{PSAT}\frac{1}{2}=\mathrm{dt}\times \ln 2\div \left(\mathrm{lnPSA}0-\mathrm{lnPSAt}\right) $$

where dt = time interval between measurement (days), PSA_0_ = initial PSA, and PSA_t_ = preoperative PSA at time *t* after the initial measurement. With use of the ultrasensitive PSA essay, an undetectable level of PSA was considered equivalent to 0.01 ng/mL.

### Postoperative follow-up

A patient was considered to have BCR when a postoperative PSA level 0.2 ng/mL above the nadir was detected after reaching a nadir PSA value of 0.1 ng/mL. Adjuvant ADT was delivered as a luteinizing hormone releasing hormone (LHRH) agonist alone, a combination of LHRH agonist with antiandrogen, or antiandrogen monotherapy, given at the discretion of the treating urologist (Y.D.C.).

CRPC was defined as three consecutive elevated PSA levels after BCR, or detection of metastasis on radiological images. In the absence of three increased PSA measurements, salvage radiotherapy on equivocal findings in radiological images was also counted as a CRPC event. Time to CRPC was determined by subtracting the date of BCR from the date of CRPC event.

### Statistical analysis

The patient cohort was classified into five groups by PSAT_½_ in 15-day intervals (non-responders [below 0: PSA increased or unchanged], ultra-rapid responders [0~15], rapid responders [15~30], intermediate responders [30~45], and slow responders [over 45]). Comparison of parameters between groups was performed using Fisher’s exact test, Mann–Whitney U test, and Kruskal-Wallis test. Survival analysis was performed by plotting Kaplan-Meier curves with a pairwise log-rank test and by constructing multivariate Cox proportional hazard regression models. All statistical analyses were performed using the Statistical Package for Social Sciences v.22.0 for Windows (SPSS; Chicago, Illinois). A *p*-value <0.05 on two-tailed test was considered statistically significant in the current study.

## Results

### Baseline characteristics

Baseline cohort characteristics are listed in Table 1. Median follow-up duration was 50.1 months (IQR 31.8–68.7). Median initial PSA was 23.2 ng/mL (IQR 11.7–48.9) and median preoperative PSA was 3.39 ng/mL (IQR 0.87–7.66). Median PSAT_½_ was 22.1 days (IQR 12.7–38.4). The number of patients assigned to each PSAT_½_ group, in order of increasing PSAT_1/2_, was 22 (6.9%), 82 (25.9%), 104 (32.8%), 40 (12.6%), and 69 (21.8%). There were 64 (20.2%) pathologic T2, 236 (74.4%) T3, and 17 (5.4%) T4 patients in the cohort, with no significant difference among groups (*p* = 0.623). There were 14 (4.4%) patients at or below Gleason score 6, 106 (33.4%) patients at Gleason score 7, and 197 (62.1%) patients at or above Gleason score 8. There was no significant difference between groups in terms of Gleason score (*p* = 0.077). There was no significant difference in duration of exposure to antiandrogen between groups, except for the slow response group, which had a median exposure of 70 (IQR 36–314) days compared to 35 (IQR 22–46) days in the non-responding group (*p* = 0.004).

**Table 1:** Baseline characteristics

		PSA half-life categories				
		Non-responder (*n* = 22)	Ultra-rapid responder (*n* = 82)	Rapid responder (*n* = 104)	Intermediate responder (*n* = 40)	Slow responder (*n* = 69)
		Median (IQR)/n (%)	Median (IQR)/n (%)	Median (IQR)/n (%)	Median (IQR)/n (%)	Median (IQR)/n (%)
Initial PSA (ng/mL)		9.6(7.7~15.2)	33.0(16.5~61.6)	23.6(12.3~47.5)	22.8(12.1~50.5)	16.0(10.2~40.9)
Immediate preoperative PSA (ng/mL)		14.3(9.6~21.1)	0.9(0.4~2.6)	2.5(0.8~6.3)	4.6(2.1~9.2)	5.4(3.7~10.4)
ADT exposure duration (days)		35.0(22.0~46.0)	51.5(37.0~78.0)	63.5(34.0~112.5)	51.5(36.0~149.5)	70.0(36.0~314.0)
PSAT_½_		−196.8(−912.9~ − 47.2)	11.3(9.9~12.9)	21.6(18.3~24.0)	33.4(32.4~38.6)	94.2(54.9~148.4)
Pathologic T stage	II	3(13.6)	13(15.9)	21(20.2)	11(27.5)	16(23.2)
	III	17(77.3)	64(78.0)	77(74.0)	29(72.5)	49(71.0)
	IV	2(9.1)	5(6.1)	6(5.8)	0(0)	4(5.8)
Pathologic Gleason score	≤6	2(9.1)	2(2.4)	3(2.9)	3(7.5)	4(5.8)
	7	5(22.7)	19(23.2)	43(41.3)	12(30.0)	27(39.1)
	≥8	15(68.2)	61(74.4)	58(58.7)	25(62.5)	38(55.1)
CRPC	No	7(31.8)	36(43.9)	61(58.7)	25(62.5)	41(59.4)
	Yes	15(68.2)	46(56.1)	43(41.3)	15(37.5)	28(40.6)
Time to CRPC (months)		13.2(6.1~21.9)	16.1(8.9~27.8)	18.8(10.0~41.2)	32.8(12.6~50.0)	18.0(6.9~43.5)

*PSA* prostate-specific antigen, *IQR* interquartile range, *ADT* androgen deprivation therapy, *CRPC* castration-resistant prostate cancer, *PSAT*_*½*_ prostate-specific antigen half-life

### Kaplan-Meier analysis for CRPC-free survival categorized by PSAT_½_ group

**Fig. 1:**
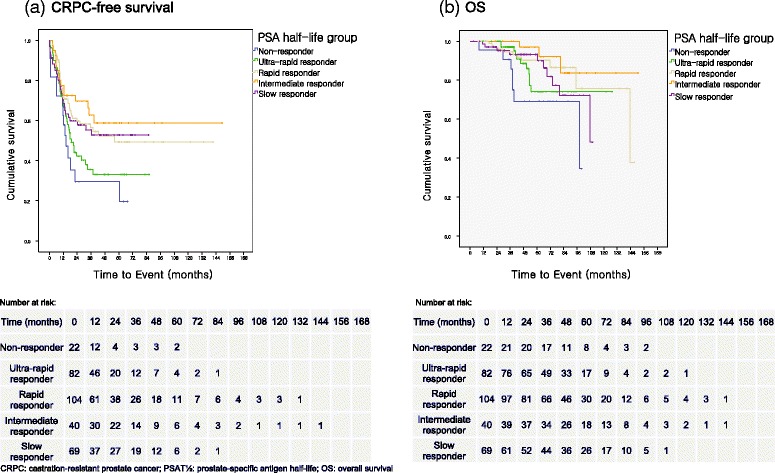
Kaplan-Meier curve for **a** CRPC-free survival and **b** OS categorized by PSAT_½_

Overall, 147 patients (46.4%) with BCR developed CRPC. Pairwise analysis of Kaplan-Meier curve (Fig. 1a) revealed that in comparison to non-response group, 5-year CRPC rate of the intermediate response group was significantly lower (37.5% vs. 63.6%, respectively; *p* = 0.007). Rapid and slow response groups also showed significantly lower rate of CRPC than non-response group (41.3%, 40.6%; *p* = 0.010, 0.041, respectively). CRPC rate in ultra-rapid responding group was similar (56.1%; *p* = 0.248) to non-responding group, and the difference with intermediate response group was also significant (*p* = 0.031). Differences between rapid or slow response groups and intermediate response group were not significant (*p* = 0.478, 0.376, respectively).

Analysis of overall mortality yielded similar results (Fig. 1b), with rapid and intermediate response groups showing improved 5-year mortality rate compared to non-responding group (7.7%, 2.5% vs. 27.3%; *p* = 0.016, 0.008, respectively). Although CRPC rate was lower in the slow response group, mortality rate difference with non-responding group was revealed to be not significant (5.8%; *p* = 0.077). Except for non-responding group, there was no significant difference in mortality between intermediate response group and ultra-rapid, rapid, and slow response groups (*p* = 0.247, 0.338, 0.419, respectively).

### Multivariate Cox proportional hazards regression analysis

To control the effects from other known prognostic factors, multivariate models were constructed (Table 2). On univariate analysis, rapid, intermediate, slow response groups (*p* = 0.011, 0.007, 0.032, respectively), Gleason score > 7 (*p* = 0.013), and pathologic T4 stage (*p* = 0.034) showed significant association with CRPC risk. Age (*p* = 0.204), initial PSA ≥20 ng/mL (*p* = 0.170) and exposure duration (*p* = 0.169) showed no significant association with CRPC. Multivariate Cox regression was performed using variables found significant in the univariate analysis. In the multivariate model, intermediate response to neoadjuvant treatment significantly predicted reduced risk for CRPC (hazard ratio [HR] 0.397, 95% confidence interval [CI] 0.191–0.823, *p* = 0.013) compared to non-responding group. Rapid response was also associated with decreased risk (HR 0.499, 95% CI 0.271–0.920, *p* = 0.026), but slow response did not show significance (*p* = 0.094) when other factors were considered. Ultra-rapid response did not affect the risk significantly in the multivariate model (*p* = 0.238) as well as in the univariate analysis. Among factors other than PSAT_½_, Gleason score > 7 (HR 1.472, 95% CI 1.018–2.128, *p* = 0.040) was also significantly associated with increased CRPC risk.

**Table 2:** Multivariate Cox regression model for CRPC and overall mortality risk with PSAT_½_ groups as parameters

		CRPC				Overall mortality			
		Univariate	Multivariate			Univariate	Multivariate		
		*p-value*	HR	95% CI	*p-value*	*p-value*	HR	95% CI	*p-value*
Age	Continuous	0.204	0.995	0.970–1.022	0.734	0.243	1.046	0.990–1.105	0.107
Initial PSA	≥20 ng/mL	0.170				0.154			
Gleason score	>7	0.013^*^	1.472	1.018–2.128	0.040^*^	0.006^*^	3.127	1.361–7.187	0.007^*^
PSAT_½_ (days)	Non-responder (below 0)	(reference)				(reference)			
	Ultra-rapid responder (0~15)	0.279	0.702	0.390–1.263	0.238	0.189	0.443	0.168–1.164	0.099
	Rapid responder (15~30)	0.011^*^	0.499	0.271–0.920	0.026^*^	0.018^*^	0.295	0.109–0.797	0.016^*^
	Intermediate responder (30~45)	0.007^*^	0.397	0.191–0.823	0.013^*^	0.011^*^	0.138	0.033–0.584	0.007^*^
	Slow responder (over 45)	0.032^*^	0.553	0.289–1.057	0.070	0.094	0.501	0.183–1.368	0.177
Pathologic T stage	Below T2	(reference)				(reference)			
	T3	0.915	0.966	0.634–1.471	0.870	0.013^*^	4.987	1.187–20.946	0.028^*^
	T4	0.034^*^	1.831	0.921–3.642	0.085	0.009^*^	9.521	1.799–50.402	0.008^*^
ADT exposure duration (days)	Continuous	0.169				0.386			

*HR* hazard ratio, *CI* confidence interval, *PSA* prostate-specific antigen, *ADT* androgen deprivation therapy, *PSAT*_*½*_ prostate-specific antigen half-life

^*^statistically significant at *p* < 0.05

Protective effect of intermediate response could be also observed in the multivariate model for overall survival. In the univariate analysis, Gleason score > 7, rapid response, intermediate response, T3 and T4 stage showed association with overall mortality (*p* = 0.006, 0.018, 0.011, 0.013, and 0.009, respectively). Age (*p* = 0.243) and the duration of exposure to ADT (*p* = 0.386) was not associated with overall survival as well. In the multivariate model, intermediate response was associated with significanly decreased mortality risk (HR 0.138, 95% CI 0.033–0.584, *p* = 0.007), followed by rapid response (HR 0.295, 95% CI 0.109–0.797, *p* = 0.016). Ultra-rapid response and slow response did not show association with overall mortality (*p* = 0.099, 0.177). In addition to Gleason score (HR 3.127, 95% CI 1.361–7.187, *p* = 0.007), high T stage increased the mortality risk (T3: HR 4.987, 95% CI 1.187–20.946, *p* = 0.028; T4: HR 9.521, 95% CI 1.799–50.402, *p* = 0.008) in our model.

### Kaplan-Meier analysis further subcategorized by Gleason score

**Fig. 2:**
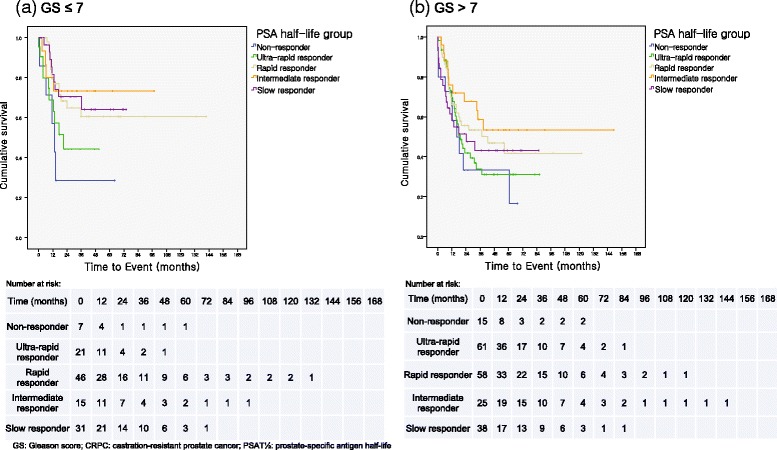
Kaplan-Meier curve of PSA half-life groups for CRPC stratified by Gleason score. **a** Gleason ≤7 stratum **b** Gleason >7 stratum

Patients were further categorized into low to intermediate Gleason score (≤7) and high Gleason score (>7) strata to analyze the influence of poor differentiation on CRPC rate in the PSAT_½_ groups (Fig. 2). When stratified according to Gleason score group, 5-year CRPC rate for non-responding patients was exceptionally higher (71.4%) compared to patients in rapid and slow response groups (32.6%, *p* = 0.020; 29.0%, *p* = 0.027; respectively) for the low to intermediate Gleason score stratum. Although CRPC rate was lower in intermediate and ultra-rapid response groups, the difference was not significant (26.7%, *p* = 0.076; 47.6%, *p* = 0.405, respectively). In the high Gleason score stratum, there were no statistically significant differences between the non-response group and other groups (*p* = 0.437, 0.174, and 0.468, for ultra-rapid, rapid, and slow response group, respectively). The only exception to this was intermediate response group, in which CRPC rate was significantly lower than in non-response group (44.0% vs. 66.7%, *p* = 0.047).

## Discussion

In the current study, we have attempted to identify patients at risk of developing CRPC by quantifying the response to ADT before the operation. By classifying according to preoperative PSAT_½_, we could identify patients at lower risk of developing CRPC from patients at high risk. Of note, our results indicate that group of patients with longer PSAT_½_ (30 to 45 days) can actually have lower CRPC rate, and that shorter PSAT_½_ is not necessarily associated with low risk of developing CRPC.

By avoiding the need to wait for PSA to achieve nadir, PSAT_½_ serves as a handy proxy for the response to ADT in the neoadjuvant setting. However, evidences so far are conflicting regarding whether the longer PSAT_½_ should be taken as a synonym for a longer remission period. In their study on PSAT_½_ during maximal androgen blockade in CRPC patients later treated with docetaxel, Lin et al. [7] concluded that PSAT_½_ > 0.5 months shortens CRPC-free survival and cancer-specific survival (HR 3.41, *p* = 0.026). On the contrary, Kim et al. [10] suggested that PSAT_½_ ≥ 0.5 months after ADT is a condition associated with increased survival after docetaxel chemotherapy. Such contradictory results may in part come from the fact that PSAT_½_ over 0.5 months represents a different subset of patients in each study, as the cohort from the former study consisted of patients with median PSAT_½_ of 0.5 (IQR 0.1–34.2) months, while the median was 0.9 (IQR 0.5–2.0) months in the latter. Additionally, in the study by Kim et al. where mean values with standard deviations were also available, the mean was 3.5 ± 9.2 months, implying that the range included negative values. Because negative half-life corresponds to PSA doubling time [13], which means a failure to achieve nadir PSA and a resistance to ADT, it would be more appropriate to categorize the patients with negative values as a separate group.

In the current study, patients with PSAT_½_ shorter than 15 days presented with increased CRPC risk comparable to that of non-response group (Fig. 1a). A number of other studies also point out that a rapid response to ADT, measured by short PSAT_½_ [10, 14] or time to nadir [15, 16], as an independent predictor of poor survival in patients with CRPC. The mechanism underlying the increase in CRPC risk for patients with steep PSA decline remains largely unknown, but postulated theories suggest that it is a result of the transcriptional effect of ADT on PSA production rather than prostate cancer cell death [16]; rapid removal of hormone-sensitive prostate cancer cells inducing an adequate environment for the growth of hormone-resistant prostate cancer cells [9]; or high probability of these subsets of patients containing cancers with low PSA production, such as cells with neuroendocrine differentiation [10] or cancer stem cells [17]. Westin et al. [18] demonstrated that the apoptotic activities are not uniformly increased in tumors responding to the androgen ablation with cellular atrophy and decreased cell proliferation rate. Considering that a decline in cell proliferation rate rapidly peaks within 7 days after castration but tumor apoptosis is found in only 30% of the patients [19], it is more likely that premature PSA decrease observed in our study was a result of halted proliferation rather than cell death. Acquisition of castration resistance in these dormant cells can result in regrowth despite the initial quick response to the androgen deprivation.

As expected, non-responders to ADT with negative PSAT_½_ showed increased CRPC risk as demonstrated by the higher CRPC rate for the non-responding group (63.6%) than the rapid response and slow response groups (Fig. 1a). These findings are in good agreement with previous literatures where an increase in PSA despite the initiation of ADT was associated with generally worse outcome. For instance, Matzkin et al. [5] showed that the median progression-free interval, for the patients whose lowest PSA level was more than 50 ng/mL, was significantly shorter than those who achieved nadir (6 vs. 12 months, *p* < 0.0001).

In our multivariate regression model, the Gleason score was the only parameter that was significantly associated with increased risk of CRPC (HR 1.472, 95% CI 1.018–2.128, *p* = 0.040) other than PSAT_½_ (Table 2). An increase in Gleason score is known to be strongly associated with higher CRPC risk. It is shown in the study by Benaim et al. [20], that each unit of increase in Gleason score increases the risk of progression into CRPC by nearly 70% (HR 1.68, 95% CI 1.3–2.1, p < 0.0001). In the low to intermediate Gleason score (≤7) patients, CRPC rate was significantly lower in the rapid and slow response groups when compared against the non-responding group, while the gap was less explicit in the high Gleason score (>7) patients, as can be seen in Fig. 2. This increase of CRPC risk in poorly differentiated cancers is similar to the finding previously documented in the study by Benaim et al. [20] in which low and intermediate Gleason score patients experienced significantly longer remissions compared to Gleason score 8 to 10 patients (52.1 vs 25.2 months, *p* = 0.0006). A notable exception to this was the intermediate response group, which also showed significant association with markedly decreased risk of CRPC in the multivariate model (HR 0.397, 95% CI 0.191–0.823, *p* = 0.013). In the high Gleason score (>7) stratum, 5-year CRPC rate (44.0%) remained lowest for the intermediate responders, especially compared to the non-response group (60.0%, *p* = 0.047). It remains to be investigated in the future studies why the group with a PSAT_½_ of 30 to 45 days maintained relatively longer remission while CRPC rate in other groups uniformly increased to a level comparable to non-responders.

A possible explanation can be that this is because patients in the current study were exposed to neoadjuvant ADT before the normal prostate tissue was completely removed by surgery. Non-malignant human prostate tissue is reported to respond to ADT with apoptosis peaking 3–4 days after castration but returning to normal levels in approximately 2 weeks [21]. Ohlson et al. [19] proposed that this effect was due to the presence of remaining epithelium and basal cells. Considering that malignant cells show pronounced (7-fold) apoptotic activity starting from day 3–4 of castration regardless of Gleason score [19], latent proliferative activity could be seen as a reflection of a higher proportion of benign prostate cells being responsible for PSA secretion, thus slowing the decline in serum PSA level weeks after ADT.

The current study has some limitation to note. While the majority of patients (74.8%) received adjuvant ADT consisting of 3-month interval LHRH agonist (Goserelin, Leuprolide and Triptorelin), a subset of patients went under combined androgen blockade with Bicalutamide (19.9%), or even Bicalutamide monotherapy (5.4%). Heterogeneous use of regimens may have interfered with maintained suppression of androgen receptor, and thus resulted in variations for PSA output. Although all patients went under routine biopsy process, its impact on PSA level cannot be determined in this study. As it is expected to cause certain level of prostatitis, raising serum PSA level, future study should consider incorporating this factor in the model. Since there is no data available regarding the serum testosterone levels of the cohort in the current study, we could not pinpoint the mechanism responsible for the observed risk increase in individual groups. Proper evaluation is necessary for the association between PSAT_½_ and the androgen activity to determine which mechanism is impaired specifically in non-responders and ultra-rapid responders.

## Conclusion

Patients with PSAT_½_ of 30 to 45 days after neoadjuvant therapy exhibited the lowest tendency to develop CRPC compared to patients with no response or ultra-rapid response who suffered high rate of adjuvant androgen deprivation therapy failure. A 1- to 3-month neoadjuvant ADT trial seems to be an effective way to identify patients who wound respond better to adjuvant ADT later, and prevent unnecessary long term ADT for patients who would not respond well to androgen deprivation.

## Additional files

Additional file 1: Raw cohort data. Raw patient parameters used in the analysis (XLSX 68 kb)

## Abbreviations

ADT: Androgen deprivation therapy
BCR: Biochemical recurrence
CI: Confidence interval
CRPC: Castration-resistant prostate cancer
HR: Hazard ratio
IQR: Interquartile range
LHRH: Luteinizing hormone releasing hormone
PSA: Prostate-specific antigen
PSAT½: Prostate-specific antigen half-life
